# Exploring the relationship between body appreciation and emotional regulation among Lebanese adults: The mediating role of psychological distress

**DOI:** 10.1371/journal.pgph.0006725

**Published:** 2026-06-18

**Authors:** Mohamad Alhourani, Nour Mhanna, Rania El Majzoub, Linda Abou-Abbas

**Affiliations:** 1 Faculty of Medical Sciences, Lebanese University, Beirut, Lebanon; 2 Department of Bioinformatics, School of Arts and Sciences, Lebanese American University, Beirut, Lebanon; 3 School of Pharmacy, Department of Biomedical Sciences, School of Pharmacy, Lebanese International University, Mazraa, Beirut, Lebanon; 4 Faculty of Medical Sciences, Neuroscience Research Center, Lebanese University, Beirut, Lebanon; 5 INSPECT-LB (Institut National de Santé Publique Epidémiologie Clinique et Toxicologie-Liban), Beirut, Lebanon; PLOS: Public Library of Science, UNITED STATES OF AMERICA

## Abstract

Body appreciation is a key component of positive body image and contributes to psychological well-being. While difficulties in emotion regulation have been linked to negative body image and psychological distress, their association with body appreciation remains understudied, particularly in non-Western populations. This study examined the relationship between emotion regulation difficulties and body appreciation among Lebanese adults and investigated whether psychological distress mediates this association. A cross-sectional online survey was conducted among Lebanese adults aged 18–65 years (N = 682; 73.9% female, 87.5% with university-level education, mean age = 27.5 years). Participants completed the Body Appreciation Scale–2 (BAS-2), the Difficulties in Emotion Regulation Scale–16 (DERS-16), and the Beirut Distress Scale–10 (BDS-10). Descriptive analyses, Spearman correlations, multiple regression, and mediation analysis were performed using IBM SPSS Statistics version 27.0, with visualization support from R. Mediation was tested using PROCESS Macro Model 4 with 5,000 bootstrap samples, controlling for age, gender, body mass index (BMI), and self-reported mental health disorders. Higher difficulties in emotion regulation were significantly associated with lower body appreciation and higher psychological distress. Psychological distress was negatively associated with body appreciation. Mediation analysis revealed that psychological distress significantly mediated the relationship between emotion regulation difficulties and body appreciation. The indirect effect was significant, indicating that greater emotion regulation difficulties were linked to lower body appreciation through increased psychological distress. Emotion regulation difficulties and psychological distress are both associated with lower body appreciation among Lebanese adults. Psychological distress was found to explain the association between emotion regulation difficulties and body appreciation. This suggests that psychological distress is an important factor to consider when examining the relationship between emotion regulation and positive body image. Further longitudinal and experimental research is warranted to better understand the nature and direction of these relationships.

## Introduction

In today’s digital era, where social media and cultural beauty ideals permeate daily life, individuals are constantly comparing themselves to unrealistic standards [1], often fueling body dissatisfaction and negative self-evaluation [2,3]. These pressures make body image a central concern in mental health research, emphasizing the importance of understanding psychological mechanisms that promote a healthier and more appreciative relationship with one’s body.

Historically, research predominantly emphasized the negative aspects of body image—such as body dissatisfaction [4], which are linked to disordered eating, low self-esteem, and mental health problems [5]. However, the emergence of positive psychology has shifted attention toward constructs that promote well-being and resilience [6]. A key concept in this paradigm is body appreciation (BA), defined as “accepting, holding favorable attitudes toward, and respecting the body” [7]. Body appreciation embodies gratitude for the body’s functionality, unconditional acceptance of imperfections, and resistance to culturally imposed beauty ideals [8]. Studies consistently show that higher body appreciation correlates with greater psychological well-being, higher self-esteem, intuitive eating, and resilience against appearance-related pressures [9,10]. Moreover, body appreciation has been associated with improved relational and sexual satisfaction and healthier lifestyle habits [11,12].

Parallel to body appreciation, emotional regulation (ER) has been identified as a fundamental process influencing mental health and self-perception. Emotional regulation refers to the ways individuals influence the experience and expression of emotions, including both adaptive strategies such as reappraisal and acceptance, and maladaptive ones like suppression and rumination [13]. Difficulties in emotion regulation have been strongly linked to a broad range of psychopathologies—including depression, anxiety, substance use, and eating disorders—highlighting ER as a transdiagnostic mechanism across mental health conditions [14,15]. Individuals who struggle to manage emotional states often rely on avoidance or self-critical patterns, which in turn amplify distress and negative body evaluations [16,17].

Emerging research indicates that body appreciation is linked to broader adaptive psychological processes and well-being indicators, including self-esteem, self-compassion, and lower psychological distress [10], suggesting potential associations with adaptive emotion-related processes. In line with this, preliminary research suggests effective emotion regulation may promote body appreciation by reducing negative affect and buffering against societal pressures [18,19].

Psychological distress (PD), encompassing symptoms of depression, anxiety, and general emotional suffering—may mediate this relationship. Individuals who have difficulty regulating their emotions tend to experience negative feelings for longer periods and react more strongly to stress, which weakens their positive self-view and makes them more self-critical [20]. Higher distress can undermine positive self-perception, whereas effective emotion regulation may reduce distress and facilitate a more accepting and appreciative attitude toward one’s body [21,22].

From an intervention perspective, increasing attention has been given to approaches that directly target these mechanisms. Interventions focusing on interoceptive awareness and embodiment aim to enhance individuals’ connection with their bodily experiences, thereby improving emotional regulation and reducing distress [23]. More recently, innovative approaches such as virtual reality have been proposed to enhance interoceptive awareness, body perception, and emotional regulation [24]. Together, these findings support the rationale for examining psychological distress as a mediator between emotion regulation and body appreciation.

In the Lebanese context, recent studies suggest that body appreciation functions as a protective psychological resource, being associated with higher optimism and lower psychological distress [25,26]. However, most research on body appreciation and emotion regulation has been conducted in Western contexts, limiting the generalizability of findings to non-Western populations [27]. Body image and body appreciation in Lebanese and broader Arab contexts are shaped within a complex sociocultural environment, where individuals may be exposed to both local norms and globalized beauty ideals [28]. Examining these constructs within this context provides an opportunity to extend existing literature and better understand their relevance across diverse populations.

The present study aims to address these gaps by examining the mediating role of psychological distress in the relationship between emotion regulation and body appreciation — a mechanism that has not been examined in Lebanese or broader Arab populations. Based on prior evidence, we hypothesize that (1) greater emotion regulation difficulties will be negatively associated with body appreciation, (2) higher psychological distress will be negatively associated with body appreciation, and (3) psychological distress will mediate the relationship between emotion regulation difficulties and body appreciation. By testing this mediation model, the study aims to provide preliminary insights that can inform culturally sensitive prevention and intervention frameworks targeting emotion regulation and body image in Lebanese populations

## Methods

### Ethics statement

This study was reviewed and approved by the Institut National de Santé Publique Epidémiologie Clinique et Toxicologie (INSPECT-LB) ethical committee that provided IRB approval on August 2025 (2025REC-006-INSPECT-07–14). The procedures of this study adhere to the ethical guidelines outlined in the Declaration of Helsinki. Informed consents were obtained electronically from all participants before their involvement. Participants were required to actively indicate their consent by selecting “Yes” before accessing the questionnaire, and they were informed of their right to withdraw at any time without any repercussions. All collected data were kept strictly confidential and anonymized to protect participants’ privacy.

### Study design and Sample

A cross-sectional design was employed. The study adopted a convenience sampling strategy due to its feasibility in reaching a broad audience. Inclusion criteria included (1) Arabic speaking participants to ensure comprehension of survey items, (2) Lebanese nationality, (3) current residence in Lebanon, (4) adults aged between 18 and 65 years old. Exclusion criteria included individuals unable to provide informed consent, unable to read and understand Arabic.

The sample size was calculated to detect a medium effect size in a multiple linear regression model examining the relationship between emotional regulation and body appreciation, while accounting for six independent variables and one mediator. Using G*Power 3.1.9.7, with a significance level of α = 0.05, a statistical power of 0.80, and an assumed medium effect size (f² = 0.15), the minimum required sample size for seven predictors was estimated to be 103 participants.

A total of 692 responses were collected, of which 10 participants were excluded for being younger than 18 years, resulting in a final sample of 682 eligible participants. Most participants were female (73.9%) and single (59.4%), with a high level of educational attainment (87.5% university degree or higher). Less than half were employed (41.1%), and the majority reported a monthly income of less than 1,000 USD (74%), while only a small fraction earned more than 2,000 USD (7.3%) (Table 1).

**Table 1:** Socio-demographic characteristics of the study participants (N = 682).

Variable	All (N = 682)
**Age (years) Mean ± SD**	27.5 ± 9.1
**Gender n (%)**	
Male	178 (26.1%)
Female	504 (73.9%)
**Marital Status n (%)**	
Single	405 (59.4%)
Married	242 (35.5%)
Divorced	24 (3.5%)
Widowed	11 (1.6%)
**Educational Level n (%)**	
No education	3 (0.4%)
Primary	5 (0.7%)
Complementary	17 (2.5%)
Secondary	60 (8.8%)
University	418 (61.3%)
Postgraduate	179 (26.2%)
**Occupation n (%)**	
Employed	280 (41.1%)
Unemployed	402 (58.9%)
**Monthly Income n (%) (n = 524)**	
<500 USD	188 (27.6%)
500–999 USD	200 (29.3%)
1000–1499 USD	67 (9.8%)
1500–1999 USD	31 (4.5%)
≥2000 USD	38 (5.6%)
**BMI (Mean ± SD)**	24 **±** 11

N, n Frequency, % Percentage, SD Standard Deviation

Data were collected over a period of 4 months, from August to December 2025, through an online questionnaire distributed via different social media platforms (e.g., Facebook, Instagram, WhatsApp groups) and community outreach (e.g., universities and community networks), targeting adults across multiple Lebanese governorates. The survey link was shared as an anonymous online form, allowing participants to complete it at their convenience. Participants were also encouraged to voluntarily share the survey link within their networks to enhance recruitment reach.

Participants were first provided with detailed information regarding the purpose and objectives of the study, the study procedures, and their rights as research participants, as outlined in the electronic informed consent form. They were also informed that participation was completely voluntary, that they could withdraw at any time without any consequences, and that all responses would remain anonymous and confidential. In addition, participants were provided with referral information for free psychological support services, including the National Mental Health Program hotline (1564), in case they experienced distress while completing the survey.

### Instruments

Data were collected using a self-administered online questionnaire, designed to be completed in approximately 10–15 minutes. The questionnaire consisted of three main sections:

1. **Socio-demographic information** – Including age, gender, marital status, educational level, occupation, and income.
2. **Clinical and psychological variables** – Including body mass index (BMI) and self-reported mental health disorder status. Participants were asked: “Have you ever been diagnosed by a healthcare professional with a mental health disorder?”
3. **Psychometric scales** –

- ***Body Appreciation Scale (BAS-2)***: Measures individuals’ acceptance and appreciation of their bodies, it is 10-item scale, Items are rated on a 5-point Likert scale ranging from *1 (Never)* to *5 (Always)*. Total Scores are calculated by averaging item responses, with higher scores indicating greater body appreciation. The original scale was developed by Tylka and Wood-Barcalow [7]. The Arabic version, validated in a Lebanese sample, demonstrated excellent psychometric properties [29]. In the current study, the BAS-2 exhibited excellent reliability (α = 0.941).
- ***The Difficulties in Emotion Regulation Scale–16* (DERS-16)** is a 16-item self-report measure assessing difficulties in recognizing, understanding, and managing one’s emotions. Items are rated on a 5-point Likert scale ranging from 1 (*almost never*) to 5 (*almost always*), with higher scores indicating greater difficulties in emotion regulation. The original scale was developed by Gratz and Roemer [20]. The Arabic version, validated in Lebanon, demonstrated excellent internal consistency and a stable factorial structure, supporting its reliability and validity in Arabic-speaking adults [30]. In this study, DERS-16 showed excellent reliability (α = 0.952).
- **Beirut Distress Scale–10** (BDS-10) is a 10-item self-report measure designed to assess levels of psychological distress specific to the Lebanese population. Items evaluate general emotional distress, including symptoms of anxiety, depression, and perceived stress. Respondents rate each item on a 5-point Likert scale ranging from 0 (*never*) to 4 (*always*), with higher scores indicating greater psychological distress. The BDS-10 has demonstrated excellent internal consistency and construct validity in Lebanese adults, supporting its use as a reliable tool for assessing distress in this cultural context [31]. The BDS-10 demonstrated good reliability (α = 0.884) in the current study.

### Statistical analysis

Statistical analyses were performed using IBM SPSS Statistics version 27 and R (version 4.3.2). SPSS was used for descriptive and inferential analyses, including correlations and regression models. R was employed to create correlation heatmaps, allowing for a graphical visualization of the relationships among study variables.

Data were screened and cleaned prior to analysis to ensure data quality. No formal attention checks or response validity filters were implemented; however, all measures were completed via an online survey platform that required full completion of all items before submission, resulting in no missing data. Potential outliers were examined using graphical methods and descriptive statistics (e.g., boxplots and inspection of standardized values), and no extreme outliers requiring exclusion were identified. Descriptive statistics were computed for all variables. Normality was assessed using the Shapiro–Wilk test and visual inspection of histograms and Q–Q plots, which indicated that all continuous variables were not normally distributed (p < 0.001). Therefore, medians and interquartile ranges (IQR) were reported for all continuous variables. Frequencies and percentages were reported for categorical variables.

Spearman correlation coefficients were used to examine associations between study variables due to non-normal distribution. Differences between participants with and without self-reported mental health disorders were assessed using the Mann–Whitney U test. Additionally, differences across categorical variables with more than two groups were examined using the Kruskal–Wallis test.

Linear regression analyses were then conducted, and the assumptions required for regression—including linearity, homoscedasticity, and absence of multicollinearity—were examined and met. Tolerance values ranged from 0.566 to 0.942, and VIF values ranged from 1.062 to 1.767, indicating no evidence of multicollinearity among predictors.

Mediation analysis was conducted using PROCESS Macro Model 4 [32] to test whether psychological distress mediated the relationship between emotion regulation difficulties and body appreciation, with ER as the predictor, PD as the mediator, BA as the outcome, and covariates including age, gender, BMI, and presence of mental health disorder.

The analysis assessed Path a (effect of ER on PD), Path b (effect of PD on BA), Path c′ (direct effect of ER on BA controlling for PD), and the indirect effect (ab) representing mediation. Bootstrapping with 5,000 resamples was used to obtain bias-corrected 95% confidence intervals for the indirect effect.

## Results

### Correlational analysis

#### Analysis of the Correlations among BA, ER, PD, age, and BMI.

**Fig 1:**
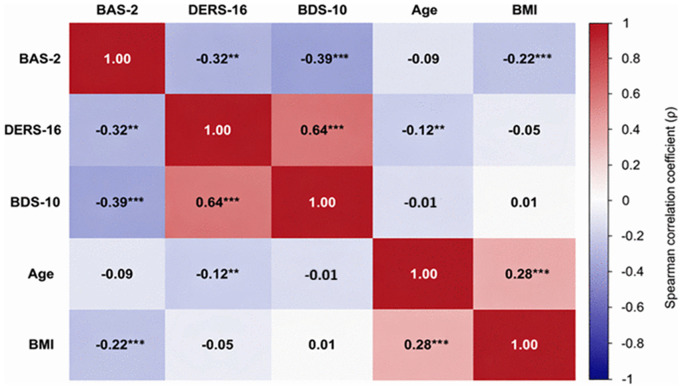
Spearman Correlation Heatmap among Study variables. Note: Values are Spearman correlation coefficients (ρ). The color intensity represents the strength of the correlation, with red indicating positive correlations and blue indicating negative correlations. A darker square represents a stronger correlation. Asterisk denote statistical Significance: *p < 0.05, **p < 0.01, ***p < 0.001. Abbreviations: BAS-2: Body Appreciation Scale-2, DERS-16: Difficulties in Emotion Regulation Scale-16, BDS-10: Body Dissatisfaction Scale-10, BMI: Body Mass Index. Bivariate Analysis of Body Appreciation by Demographic Characteristics and self-reported Mental Health Disorders.

Fig 1 presents a correlation heatmap illustrating the relationships among BA, ER, PD, age, and BMI. The blue color indicated a negative correlation, while the red color indicated a positive correlation. A darker square represents a stronger correlation.

BA was significantly and negatively correlated with ER (r = –0.32, p < 0.01) and PD (r = –0.39, p < 0.001). ER and PD were strongly positively correlated (r = 0.64, p < 0.001). Age was weakly negatively correlated with ER (r = −0.12, p = 0.003), but was not significantly correlated with BA (r = –0.09, p > 0.05) and PD (r = −0.01, p = 0.760). BMI was negatively associated with BA (r = –0.22, p < 0.001) and positively associated with age (r = 0.28, p < 0.001), while no significant correlation was observed with ER (r = −0.05, p = 0.156).

Bivariate analyses examining the association between body appreciation scores and demographic characteristics showed no statistically significant differences (Table 2).

**Table 2:** Bivariate Analysis of Body Appreciation (BAS-2) by Demographic Characteristics.

Variable	Category	n	Median (IQR)	Test static (U/H)	P-value
**Gender**	Male	178	39 (10)	44791.5	0.977
	Female	504	39 (10)		
**Occupation**	Employed	280	38.5 (9)	52168	0.104
	Unemployed	402	39 (11)		
**Marital Status**	Married	242	38 (11)	7.177	0.066
	Divorced	24	39 (5)		
	Single	405	39 (11)		
	Widowed	11	38.5 (7)		
**Income (USD)**	<500	188	39 (11)	5.562	0.234
	500–999	200	39 (10)		
	1000–1499	67	38 (12)		
	1500–1999	31	38 (6)		
	≥2000	38	40.5 (14)		
**Educational Level**	No education	3	29(-)	9.951	0.088
	Primary	5	23 (-)		
	Complementary	17	39 (11)		
	Secondary	60	37 (14)		
	University	418	39 (10)		
	Postgraduate	179	38.5 (9)		

*Note: n Frequency, IQR Interquartile. Group comparisons were performed using the Mann–Whitney U test for two-group variables and the Kruskal–Wallis H test for variables with more than two groups. P-value less than 0.05 is considered significant.*

Bivariate analyses indicated significant differences in BAS-2 scores according to participants’ self-reported mental health disorders. Participants reporting any mental health disorder had significantly lower body appreciation compared to those without a disorder (Median = 40 vs. 37; p-value = 0.001). Specifically, individuals with depression (Median = 36.5; p-value = 0.001) or anxiety (Median = 38; p-value = 0.006) also reported significantly lower BAS-2 scores. No statistically significant differences were observed for bipolar disorder (p-value = 0.156), OCD (p-value = 0.189), or eating disorders (p-value = 0.119) (Table 3).

**Table 3:** Bivariate Analysis of Body Appreciation by self-reported Mental Health Disorders.

Variable	Category	n	Median (IQR)	Test Statistic (U)	P-value
Any Mental Health Disorder	No	558	40(11)	25019.5	0.001*
	Yes	124	37 (15)		
Obsessive-Compulsive Disorder	No	681	39(11)	82.5	0.189
	Yes	1	–		
Depression	No	620	39(11)	13121.5	0.001*
	Yes	62	36.5(16)		
Anxiety	No	607	39(11)	18377.5	0.006*
	Yes	75	38(13)		
Bipolar Disorder	No	677	39(11)	1071.0	0.156
	Yes	5	38(22)		
Eating Disorders	No	676	39(11)	1280.0	0.119
	Yes	6	31.5(18)		

*Note: N, n Frequency. Group comparisons were performed using the Mann–Whitney U test for two-group variables and the Kruskal–Wallis H test for variables with more than two groups. P-value less than 0.05 is considered significant.*

### Multivariable analysis

A multiple linear regression analysis was conducted to examine the associations of DERS-16 BDS-10, age, gender, BMI, and the presence of a self-reported mental health disorder with BAS-2. The overall model was statistically significant, F (6, 674) = 30.10, p < 0.001, and explained 21.1% of the variance in BA scores (R² = 0.211; adjusted R² = 0.204). Higher scores on DERS-16 were significantly associated with lower BA (Unstandardized β= −0.073, p = 0.005). BDS-10 was also negatively associated with BA (Unstandardized β = −0.311, p < 0.001). Additionally, higher BMI was associated with lower BA (Unstandardized β = −0.328, p < 0.001), and participants reporting a mental health disorder had significantly lower BA scores (Unstandardized β = −1.562, p = 0.049). Age and gender were not significantly associated with BA in the adjusted model (Table 4).

**Table 4:** Multiple Linear Regression Predicting Body Appreciation (BAS-2).

Predictor	Unstandardized β	SE	β	p-value	95% CI
DERS-16	−0.073	0.026	−0.125	0.005	−0.124, −0.022
BDS-10	−0.311	0.051	−0.277	<0.001	−0.411, −0.210
Age (years)	−0.047	0.034	−0.051	0.167	−0.114, 0.020
Gender (female)	1.322	0.677	0.069	0.051	−0.008, 2.652
BMI (kg/m²)	−0.328	0.059	−0.200	<0.001	−0.444, −0.212
Self-reported Mental health disorder (yes)	−1.562	0.792	−0.072	0.049	−3.118, −0.006

*Model fit: R² = 0.211; Adjusted R² = 0.204; F(6, 674) = 30.10; p < 0.001; Durbin–Watson =* 1.80

*Note: Bootstrapped estimates (5,000 resamples). Gender coded as 0 = male, 1 = female.*

### Mediation analysis

A mediation analysis was conducted using PROCESS Macro Model 4 [32]to examine whether BDS-10 mediated the association between ER and BA, controlling for age, gender, BMI, and the presence of a self-reported mental health disorder.

In the total effect model, DERS-16 was significantly associated with BA (*B* = −0.168, *p* < 0.001). In the mediator model, higher difficulties in emotion regulation were significantly associated with higher psychological distress (*B* = 0.306, *p* < 0.001). In the outcome model, BDS-10 was significantly associated with BA (*B* = −0.311, *p* < 0.001). The direct effect of DERS-16 on BA remained statistically significant but was attenuated after including the mediator (*B* = −0.073, *p* = 0.005).

The indirect effect of DERS-16 on BA through BDS-10 was statistically significant (*B* = −0.095, SE = 0.017), with a 95% bias-corrected bootstrap confidence interval that did not include zero. The indirect effect accounted for 56.5% of the total effect (Table 5).

**Table 5:** Mediation effect of BDS-10 in the Relationship Between DERS-16 and BAS-2.

Path	Pathway	Effect	BootSE	p-value	95% CI
**Total effect**	ER → BA	−0.168	0.021	<0.001	−0.210, −0.126
**Direct effect**	ER → BA	−0.073	0.026	0.005	−0.124, −0.021
**Indirect effect**	ER → PD → BA	−0.095	0.017	—	−0.129, −0.062
**Path a**	ER → PD	0.306	0.016	<0.001	0.275, 0.337
**Path b**	PD → BA	−0.311	0.051	<0.001	−0.411, −0.210

*Bias-corrected bootstrapping with 5,000 resamples. Models adjusted for age, gender, BMI, and presence of a mental health disorder. Abbreviations: BootSE: Bootstrap Standard Error, 95%CI: 95% Confidence Interval.*

**Fig 2:**
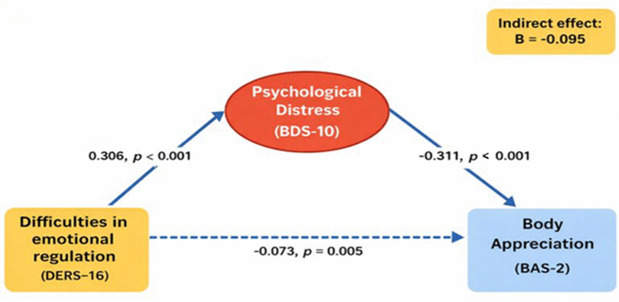
Mediation model illustrating the indirect effect of difficulties in emotion regulation (DERS-16) on body appreciation (BAS-2) through psychological distress (BDS-10).

Fig 2 illustrates the mediation model where PD mediates the relationship between ER and BA. The model shows the direct effect of emotion regulation difficulties on body appreciation, as well as the indirect effect through psychological distress. Standardized path coefficients and confidence intervals are depicted for each pathway, highlighting the magnitude and significance of the relationships.

## Discussion

The present study examined the associations between emotion regulation, psychological distress, and body appreciation among Lebanese adults, and tested psychological distress as a mediating mechanism in this relationship. Overall, the findings provide support for the proposed model, indicating that difficulties in emotion regulation are associated with lower body appreciation both directly and indirectly through increased psychological distress.

Consistent with prior research, greater difficulties in emotion regulation were strongly associated with higher psychological distress [30–32]. Individuals who struggle to regulate their emotions may experience heightened emotional reactivity and prolonged negative affect, increasing vulnerability to stress, anxiety, and depressive symptoms. The strength of this association in the present sample underscores the central role of emotion regulation in psychological functioning across cultural contexts.

Psychological distress was, in turn, negatively associated with body appreciation. This finding aligns with growing evidence that elevated distress undermines positive body image [10, 11, 20, 33] by fostering maladaptive cognitive and emotional processes, including rumination, self-criticism, and attentional bias toward perceived bodily flaws [34–36]. From a cognitive–behavioral perspective, psychological distress may intensify negative self-schemas and reduce emotional resources necessary for body acceptance and self-compassion. The present results extend prior work by demonstrating that psychological distress is not only linked to negative body image but also plays a significant role in diminishing positive body image constructs such as body appreciation.

Importantly, mediation analyses revealed that psychological distress mediated the relationship between emotion regulation difficulties and body appreciation. This suggests that individuals with greater difficulties regulating emotions may experience lower body appreciation partly because these difficulties contribute to elevated psychological distress. At the same time, the persistence of a significant direct effect suggests that emotion regulation difficulties are associated with body appreciation through additional pathways independent of distress. The model explained 21% of the variance in body appreciation (R² = 0.211), indicating a small-to-moderate effect size commonly observed in psychosocial research where multiple unmeasured factors are expected to contribute to body image outcomes.

These pathways may include reduced self-compassion, increased emotional avoidance, or heightened internalization of sociocultural appearance ideals [37,38]. Together, these findings support theoretical models proposing that emotion regulation shapes body image not only through emotional distress but also through broader self-evaluative and regulatory processes [39].

The absence of gender differences may reflect shifting sociocultural norms and increasing appearance-related pressures across genders. Similarly, the limited role of sociodemographic variables suggests that internal psychological processes may play a more prominent role than demographic characteristics in shaping body appreciation.

From a cultural perspective, this study contributes to the limited literature on positive body image in Arab and Middle Eastern contexts. Lebanon represents a unique sociocultural setting characterized by strong social comparison pressures, media exposure, and evolving beauty standards influenced by both Western and local norms. The findings provide preliminary evidence that the mechanisms linking emotion regulation, psychological distress, and body appreciation observed in Western samples may also be relevant in this context, although this should be interpreted with caution and confirmed in more representative Lebanese samples. This highlights the potential cross-cultural applicability of emotion regulation and positive body image frameworks, while underscoring the importance of culturally sensitive interventions.

### Strengths and limitations

To our knowledge, this study is the first to examine the relationship between emotion regulation and body appreciation in a Lebanese adult sample predominantly young and educated, addressing a gap in the literature on culturally relevant body image research. Additionally, the use of reliable and validated scales (DERS-16, BDS-10, BAS-2) strengthens the validity and comparability of the findings with prior studies. However, the present study has several limitations. First, the cross-sectional design precludes causal inferences, limiting the ability to determine the temporal relationships among emotion regulation, psychological distress, and body appreciation. Second, all measures were self-reported, which may introduce response and social desirability biases. Individuals with neurological disorders, psychotic conditions, or cognitive impairments were not specifically screened for and therefore were not excluded from participation; eligibility was based on self-reported ability to provide informed consent and complete the online questionnaire. Psychiatric conditions were also self-reported; clinical verification was not feasible in this survey design, which may introduce misclassification bias, as participants may underreport, overreport, or inaccurately identify their diagnoses. This limitation may affect the precision of grouping and the interpretation of associations involving mental health status. Third, the sample was predominantly female, relatively young, and highly educated, which may limit the generalizability of the findings to the broader Lebanese adult population. Additionally, gender was assessed dichotomously (male/female), and the absence of non-binary or “prefer not to say” options limits representation of gender-diverse individuals. Therefore, the results should be interpreted with caution and may not be representative of all Lebanese adults. Fourth, Additional unmeasured factors, such as the presence of comorbid symptoms and the duration or severity of psychiatric symptoms, were not assessed in the present study. This may limit a more comprehensive understanding of participants’ clinical profiles and could influence the observed associations. Additionally, although multiple covariates were controlled for, unmeasured factors—such as media exposure, physical activity, and chronic health conditions—may also influence attitudes toward one’s body. Future research should employ longitudinal or experimental designs to clarify causal pathways and explore additional mediators (e.g., self-compassion or sociocultural internalization), and test intervention-based models.

### Clinical and public health implications

The findings of this study have important implications for clinical practice and public health interventions aimed at promoting positive body image and psychological well-being. Psychological interventions designed to enhance body appreciation should integrate strategies that target both emotion regulation capacities and psychological distress, as addressing distress alone may be insufficient for fostering sustainable improvements in body image. Approaches such as mindfulness-based interventions, acceptance-based and emotion-focused therapies, and cognitive-behavioral techniques may be particularly effective in strengthening adaptive emotion regulation skills while reducing emotional distress. Given the sociocultural context of Lebanon, it is essential that such interventions be culturally sensitive and responsive to local body image norms, values, and social pressures to ensure relevance and effectiveness. Recent advances in immersive technologies, such as Mixed Reality (MR) and Virtual Reality (VR), have been increasingly explored as adjunct tools in psychological interventions [40]. Interactive 3D environments can reduce cognitive load and foster positive emotional engagement [41]. Applied to body image context, VR could be used to support therapeutic work by enhancing engagement and providing opportunities to practice cognitive and emotional skills within simulated situations. At the public health level, preventive initiatives could include educational and awareness campaigns that promote emotional awareness, adaptive coping strategies, and positive body appreciation, particularly within schools and community settings. Furthermore, healthcare providers and policymakers should recognize the role of psychological processes in shaping body image and incorporate mental health support into holistic health promotion programs, thereby addressing body image concerns as a public mental health priority rather than an isolated clinical issue.

## Conclusion

In conclusion, higher difficulties in emotion regulation were associated with lower body appreciation and higher psychological distress among Lebanese adults. Psychological distress mediated the relationship between emotion regulation difficulties and body appreciation, indicating that individuals with greater emotion regulation difficulties tend to experience higher psychological distress, which is linked to lower body appreciation. These findings highlight the importance of addressing both emotion regulation skills and psychological well-being in efforts to promote positive body image. Future longitudinal and experimental studies are warranted to clarify the directionality and causal relationships among these constructs.

## Supporting information

S1 Data Data. (XLSX)
