# Supplementary material for: Exploring the relationship between body appreciation and emotional regulation among Lebanese adults: The mediating role of psychological distress
Source: PLOS Glob Public Health. 2026 Jun 18;6(6):e0006725. doi: 10.1371/journal.pgph.0006725 (PMC13278471; doi:10.1371/journal.pgph.0006725)
Supplement: Attachment — Submitted filename: Response to Reviewers.docx [file pgph.0006725.s003.docx]

April 28, 2026

Dear Dr Zimmerman,

We are pleased to resubmit the revised version of Manuscript entitled: “***Exploring the Relationship between Body Appreciation and Emotional Regulation Among Lebanese Adults: The Mediating Role of Psychological Distress”***

**Ref:** Submission ID

We would like to thank the editor and the reviewer for their very helpful comments. We have carefully addressed all the comments. We hope our revision has improved the paper to a level of their satisfaction. Our responses are in italics and are prefaced by “Author response.” Corresponding changes are found in the revised file. Please find attached our revised paper, and below is a summary of how we responded to the comments. My coauthor and I appreciate the opportunity to resubmit and are excited about the possibility of publication.

Best regards,

**Reviewer #1**:

- Introduction: please, foster your background with further details regarding psychological constructs (see: Ongaro et al., 2021) and related interventions. For example, what about how to address these points? Please, see: Sebri et al., 2020; 2023.

**Author Response:** *Thank you for this suggestion. We have added a paragraph integrating Sebri et al. (2020; 2023) to include an intervention-oriented perspective, emphasizing approaches such as interoceptive awareness in promoting positive body image.*

-Second, please, structure your hypothesis and goals better

***Author Response:*** *Thank you for this helpful comment. We have revised the final section of the Introduction to improve the clarity and structure of the study aims and hypotheses. Specifically, we have reformulated the hypotheses into clearly defined, directional statements, including the associations between emotion regulation difficulties, psychological distress, and body appreciation, as well as the mediating role of psychological distress.*

- Methods: the recruitment process should be better explained

***Author Response:*** *We thank the reviewer for this comment. We have revised the Methods section to provide a clearer and more detailed description of the recruitment process.*

- Discussion: I would suggest proposing new approaches to this topic. As known, new technologies are currently an innovative, effective approach that could be applied, with benefits and limits. Please, discuss this point, considering your research study and findings (see: Triberti et al., 2021).

 ***Author Response:*** *Thank you for your comment. In the revised manuscript, we now highlight that these technologies may support the delivery of evidence-based techniques.*

**Reviewer #2:** The manuscript addresses a timely and culturally relevant question: whether psychological distress mediates the association between emotion regulation difficulties and body appreciation in Lebanese adults. The topic is well-positioned within the growing literature on positive body image and contributes to the understudied non-Western context. The study design is appropriate, the sample size exceeds the a priori power calculation by a wide margin (N = 682 vs. 103 required), and the statistical approach (PROCESS Model 4 with bootstrapping) is sound. The validated instruments (BAS-2, DERS-16, BDS-10) are culturally appropriate, all having Arabic-language validation studies conducted in Lebanese samples. The writing is generally clear, and the structure follows standard IMRAD format.

However, several methodological and presentational issues must be addressed before the manuscript can be recommended for publication. These range from a substantive citation error and inconsistencies in reported statistics to gaps in the methodological description and underexplored aspects of the discussion. None of these issues are fatal, but they require careful attention.

- Misattribution of the BAS-2 source citation: At line 167, the manuscript describes the BAS-2 and cites reference [20] (Gratz & Roemer, 2004) as the source. Reference [20] is the original development paper for the DERS, not the BAS-2. The correct citation for the BAS-2 is reference [7] (Tylka & Wood-Barcalow, 2015). This error also appears at line 173, where [20] is correctly cited for the DERS. The dual citation of the same reference for two different instruments is confusing and must be corrected: line 167 should cite [7], not [20].

***Author Response:*** *Thank you for this helpful observation. We appreciate the reviewer’s careful reading. The citation at line 167 has been corrected to refer to Tylka & Wood-Barcalow (2015) for the BAS-2, while Gratz & Roemer (2004) has been retained appropriately for the DERS.*

-Discrepancy in Table 4 (Mental Health Disorder medians)

Table 4 reports that participants with “Any Mental Health Disorder = No” have a median BAS-2 of 37 (IQR 15), while those with “Yes” have a median of 40 (IQR 11). This is counterintuitive — it suggests that individuals reporting a mental health disorder have higher body appreciation than those without one. However, the text at lines 277–278 states the opposite: “Participants reporting any mental health disorder had significantly lower body appreciation compared to those without a disorder (Median = 40 vs. 37; p-value = 0.001).” One of these is incorrect. Given that the text assigns Median = 40 to those without a disorder and 37 to those with one, the table rows for “No” and “Yes” appear to be inverted. The authors must verify the raw data and correct either the table or the text.

***Author Response:*** *Thank you for pointing this issue. We have re-checked the raw dataset and statistical outputs to verify the reported values. The discrepancy was due to an inversion in Table 4 labels for the “No” and “Yes” groups. The table has now been corrected. Accordingly, participants without a mental health disorder show higher body appreciation (Median = 40) compared to those reporting a disorder (Median = 37), as correctly stated in the text.*

-Convenience sampling and generalizability

The sample is 73.9% female, 87.5% university-educated, and predominantly young (mean age 27.5). The convenience sampling strategy via social media introduces considerable selection bias. While the authors acknowledge this in the Limitations section, the Discussion interprets results as if they generalize to “Lebanese adults” broadly. The cultural generalizations about Lebanon (lines 387–393) should be tempered, and the title itself (“Among Lebanese Adults”) could include a qualifier (e.g., “predominantly young and educated”). The Discussion should explicitly state that the demographic skew limits inferences about the broader Lebanese population.

***Author Response:*** *We thank the reviewer for this important observation. We have revised the Discussion to temper the generalization of the findings, particularly with regard to the broader Lebanese population. The sample’s demographic skew (predominantly young, highly educated, and female) is acknowledged, and we have clarified that the results may not fully generalize to all Lebanese adults (refer to page17. Line 403/ 417-420). However, we retain the title "Among Lebanese Adults" as the study focuses on a Lebanese sample, which remains relevant despite the demographic composition.*

-Covariates inconsistency between regression and mediation
The multiple regression model (Table 5) includes six predictors: DERS-16, BDS-10, age, gender, BMI, and self-reported mental health disorder. However, the mediation analysis (lines 215–217) states that covariates included “age, gender, BMI, presence of mental health disorder, marital

status, education, occupation, income, and governorate.” This means the mediation model controlled for five additional covariates (marital status, education, occupation, income, governorate) that were absent from the regression model. The authors should explain why the two models used different covariate sets, or ideally harmonize them. If the covariates were intentionally different, justification is needed.

***Author Response****: Thank you for your comment. We would like to clarify that the covariates used in both the regression and mediation models were the same set: age, gender, BMI, presence of mental health disorder, and self-reported mental health disorder. These covariates were selected based on their statistical significance in the bivariate analysis and those theoretically proven by literature to affect BA. We have updated the manuscript to ensure there is no confusion.*

-Missing non-binary or non-response gender option Gender is coded dichotomously (male/female). The manuscript does not mention whether a non-binary or “prefer not to say” option was offered. Given that body image research is increasingly sensitive to gender diversity, the authors should clarify the gender categories available in the survey and acknowledge this as a limitation if no non-binary option was offered.

***Author Response:*** *The gender options provided in the survey were dichotomous (male/female), and we did not include a non-binary or "prefer not to say" option. While gender diversity outside the male/female binary is increasingly recognized in global research, the non-binary or "prefer not to say" options were not included in the current study due to the specific cultural and societal norms of Lebanon.*

-Typo issues: “Tittle” appears at line 1 of the manuscript body. Should be “Title.”

***Author Response:*** *Thank you for pointing this out. The typo has been corrected as suggested*.

-Sentence fragment at lines 115–118: “The present study aims to address these gaps by examining the mediating role of psychological distress in the relationship between emotion regulation and body appreciation has not been examined in Lebanese or broader Arab populations.” This sentence conflates two clauses and is grammatically broken. Suggested revision: “The present study aims to address these gaps by examining the mediating role of psychological distress in the relationship between emotion regulation and body appreciation — a mechanism that has not been examined in Lebanese or broader Arab populations.”

***Author Response:*** *Thank you for this helpful suggestion. The sentence has been revised accordingly to improve clarity.*

-Lowercase “higher” at line 108: “makes them more self-critical [20]. higher distress can undermine…” — “Higher” should be capitalized as it begins a new sentence.

***Author Response:*** *Thank you for noting this. The capitalization has been corrected.*

-Missing space before citation at lines 86–87: “respecting the body”[7] should be “body” [7].
***Author Response:*** *The spacing has been corrected.*

-Correlation report inconsistency for BDS-10 and Age: lines 267–268 report the age–BDS-10 correlation as r = −0.11, p < 0.05, but Figure 1 (heatmap) shows this value as −0.01. The manuscript text and the figure are inconsistent. The authors must verify which value is correct.

***Author Response:*** *We thank the reviewer for identifying this inconsistency. We have rechecked the analyses and confirmed that the correct correlation between age and BDS-10 is r = −0.01, p = 0.760.*

-BMI correlation with DERS-16: Figure 1 shows BMI–DERS-16 correlation as −0.05, but this is not reported in the text. For completeness, it should be noted.

***Author Response:*** *We thank the reviewer for this helpful suggestion. We have revised the manuscript to include the BMI–DERS-16 correlation in the text for completeness.*

-Also, the text (lines 267–269) reports BMI correlated with BAS-2 as r = −0.219, while Figure 1 shows −0.22 — this is consistent but the text should round to two decimal places uniformly throughout.

***Author Response:*** *We thank the reviewer for this suggestion. We have standardized rounding to two decimal places*.

-Monthly income data: Table 1 reports income data for n = 524, meaning 158 participants (23.2%) did not report income. This missingness is not discussed. The authors should mention this and clarify whether these participants were excluded from income-related analyses.

***Author Response:*** *We thank the reviewer for this important observation. We have clarified that monthly income data were missing for 158 participants (23.2%) and that this missingness was handled by excluding these cases pairwise only in analyses involving income, while all other analyses were conducted using available data.*

-Ratio of mediation: The manuscript reports partial mediation but does not report the proportion of the total effect mediated (indirect/total = 0.095/0.168 = 56.5%). Including this ratio would strengthen the interpretation.

***Author Response:*** *We thank the reviewer for this helpful suggestion. We have now included the proportion of the total effect mediated in the Results section.*

-Effect size interpretation: R-squared = 0.211 means the model explains about 21% of BAS-2 variance. While the authors report this, they do not discuss whether this is a meaningful or modest amount for a model with six predictors. Contextualising this against comparable studies would be helpful.

***Author Response:*** *We thank the reviewer for this insightful comment. We have now expanded the Discussion to contextualize the R-squared value (0.211), noting that the model explains approximately 21% of the variance in body appreciation, which is generally considered a small-to-moderate effect size in psychological research, particularly for multifactorial constructs such as body image.*

-Figure 2 inconsistency: The mediation path diagram shows Path b as p = 0.001, but Table 6 reports p < 0.001. These should be harmonised.

***Author Response:*** *Corrected*

Bibliographic Issues

-Reference [20] misattribution: Gratz & Roemer (2004) is cited for BAS-2 at line 167, but describes the DERS. Must cite [7] instead.

***Author Response:*** *Corrected.*

-Reference [4]: Author surname is misspelled as “TyLka” — should be “Tylka.” Page range is listed as pp. 56–58 but likely spans pp. 56–64 (confirm against the physical volume). Chapter title is absent.

***Author Response:*** *We thank the reviewer for this comment. We have verified the reference and confirm that the chapter title (“Positive psychology perspectives on body image”) is included. We have nonetheless corrected the author’s surname to “Tylka” and updated the page range to ensure consistency with the original source.*

-Reference [5] (DSM-5): Citation is incomplete — missing full title (Diagnostic and statistical manual of mental disorders, 5th ed.), publisher (American Psychiatric Publishing), and DOI (10.1176/appi.books.9780890425596).

***Author Response:*** *The DSM-5 reference has been completed by adding the full title, publisher, and DOI to ensure accurate and complete citation.*

-Reference [29] (Hayes, PROCESS macro): The citation is incomplete. Hayes (2022) refers to Introduction to Mediation, Moderation, and Conditional Process Analysis (3rd ed., Guilford Press). The URL alone is insufficient; full book citation should be provided.

***Author Response:*** *We thank the reviewer for this comment. We have revised the reference by providing the full book citation for Hayes (2022) (now its reference [32])*

Reference [21] (Levy et al.): Year cited as 2025, but CrossRef records online publication in 2024. Verify print issue date.

***Author Response:*** *We thank the reviewer for this observation. We have verified the publication details and updated the reference year to reflect the correct online publication date (2024).*

-References [14], [17]: Multiple co-authors omitted (Nolen-Hoeksema & Schweizer for [14]; five co-authors for [17]). While Vancouver-style numeric citations can abbreviate, PLOS journals use a full author list. Verify compliance with journal citation guidelines.

***Author Response:*** *Corrected*

Recommendation: Minor Revision.

The manuscript makes a relevant contribution to the body image literature in a non-Western context. The study is methodologically sound in its core design, and the mediation analysis is well-executed. However, the citation error (Major Issue 2.1), the likely table inversion (Major Issue 2.2), the covariate inconsistency between models (Major Issue 2.4), and the correlation discrepancy between text and Figure 1 (Minor Issue 3.5) must be resolved before publication. These appear to be correctable errors rather than fundamental design flaws. Once addressed, the paper will be suitable for publication in PLOS Global Public Health.

***Author’s response:*** *We sincerely thank the reviewer for their thoughtful and constructive feedback on our manuscript. We appreciate the recognition of the study’s contribution to the body image literature in a non-Western context and its methodological rigor. All the points raised have been carefully considered and addressed in the revised version of the manuscript. Specifically, we have corrected the citation error, resolved the table inversion, ensured consistency in covariates across models, and clarified the discrepancy between the reported correlations and Figure 1. We believe these revisions have strengthened the clarity and accuracy of the manuscript.*

**Reviewer #3:** The paper investigates the association between emotion regulation difficulties and body appreciation among Lebanese adults, and examines the mediating role of psychological distress. The cultural context is valuable, and the manuscript is generally clear. To further improve readability, some reorganization and clarifications are suggested.
Below are my comments:

1) Page 4: In the “Introduction” section:
- After introducing emotion regulation for the first time, please indicate the abbreviation (ER) to create a clear reference for its later use in the text.

***Author Response: Thank you for noting this. The abbreviation (ER) has been added at first mention.***

- “Despite growing evidence linking emotion regulation to negative body image outcomes, little is known about its relationship with positive body image.” A meta-analysis reported positive associations between body appreciation and adaptive emotion regulation (see Linardon et al., 2022); it may be useful to include this data and reference.
Reference:
Linardon, J., McClure, Z., Tylka, T. L., & Fuller-Tyszkiewicz, M. (2022). Body appreciation and its psychological correlates: A systematic review and meta-analysis. Body Image, 42, 287–296.

***Author Response: Thank you for this valuable suggestion. We have revised the Introduction accordingly by incorporating findings from Linardon et al. (2022) and clarifying the interpretation of the evidence. Specifically, we have avoided overstating a direct association between emotion regulation and body appreciation, and instead framed the evidence in terms of broader adaptive psychological processes and potential emotion-related links.***

- The statement that “most existing research on body appreciation and emotion regulation has been conducted in Western contexts” is relevant. In this regard, it may be useful to further elaborate, already in the Introduction, on how body image and body appreciation are conceptualized within the Lebanese or broader Arabic context, in order to better support the rationale for the study. This would also facilitate a clearer link with the discussion of cultural aspects later in the manuscript.

***Author Response: Thank you for this valuable suggestion. We have added a brief paragraph in the Introduction to acknowledge the potential influence of sociocultural factors on body image in Lebanese and broader Arab contexts. Given that cultural variables were not directly assessed in the present study, this addition was framed cautiously to provide contextual background without overstating empirical conclusions.***

2) Page 6: The “Methods” section appears somewhat fragmented.

It may be useful to consolidate and streamline sample-related information into a single paragraph, “Sample”, including the sample size (with the sample size calculation reported in a footnote), online data collection procedures, recruitment (please provide examples of social media platforms and community outreach), inclusion and exclusion criteria, and self-reported mental health disorders. A brief description of how participants received study information, provided informed consent, and that participation was voluntary would also be helpful. Referring to Table 1 for detailed socio-demographic information could avoid listing all variables in the text.

***Author Response: Thank you for this helpful suggestion. We have restructured the Methods section to improve clarity and coherence by consolidating all sample-related information into a single “Sample” subsection, including the sample size, online data collection procedures, recruitment (with examples of social media platforms and community outreach), inclusion and exclusion criteria, and self-reported mental health disorders. Additionally, we have provided a brief description of how participants received study information, provided informed consent, and that participation was voluntary. We have also referred to Table 1 for detailed socio-demographic information, as suggested. Regarding the sample size calculation, we understand the importance of transparency. Instead of placing this in a footnote, we have included the sample size calculation directly in the text of the Methods section to ensure accessibility and clarity for readers.***

3) Page 7: The “Data collection” section could be renamed “Instruments” and structured to list the selected measures.

For each instrument, please report the original validation reference followed by the Arabic validation reference, and include the internal consistency in the current sample.

For the “clinical and diagnosed mental health variables,” please specify the exact questions used.

As Table 2 appears to report similar information, this may be redundant, and its removal could be considered.

***Author Response: The Data collection section has been renamed “Instruments” and restructured accordingly. For each measure, we now report the original validation, Arabic validation, and internal consistency in the current sample. We also clarified the exact question used for the self-reported mental health disorder variable. Additionally, we have removed Table 2.***

4) Page 8: “Statistical Analysis” section: before describing how the analyses were performed, please indicate whether all measures were completed and whether any validity checks (e.g., response time, attention filters) were used to identify careless responders or bots.

***Author Response: We thank the reviewer for this valuable suggestion. we now state that all measures were completed online requiring full completion of all items before submission, resulting no missing data. We also clarified that no formal attention checks or response validity filters were implemented, while indicating that the dataset was screened for completeness prior to analysis.***

- Please also indicate how potential outliers were examined and handled.

***Author Response: we added that potential outliers were examined using descriptive statistics and graphical inspection (e.g., boxplots and standardized values), and no extreme outliers requiring exclusion were identified.***

- The statement “for continuous variables that were approximately normally distributed” is somewhat vague; please clarify how normality was assessed (e.g., Shapiro–Wilk test).

***Author Response: We acknowledge that mean and standard deviation were initially reported in Tables 1 and 2 by mistake. This has now been corrected, and all continuous variables are consistently reported as median (IQR) to align with the observed data distribution. We have also clarified this approach in the manuscript text within the Statistical Analysis section.***

- Please report Tolerance and VIF values to address potential multicollinearity.

***Author Response: We thank the reviewer for this important suggestion. We have addressed the potential issue of multicollinearity by calculating and reporting tolerance and Variance Inflation Factor (VIF) values for all predictors included in the regression model. The results indicated that all tolerance and VIF values were within acceptable ranges, suggesting no evidence of multicollinearity among the independent variables.***

- Before describing the mediation analysis, please specify the types of correlations computed and the statistical tests used to compare participants with and without mental health disorders.

***Author Response: We clarified in the Statistical Analysis section that Spearman correlations were used due to non-normality, and that group differences (mental health disorder: yes/no) were tested using the Mann–Whitney U test.***

- Throughout the manuscript, referring to constructs rather than instrument names may improve readability, both in the text and in the tables.

***Author Response: We thank the reviewer for this suggestion. We revised the manuscript to refer to constructs rather than instrument names to improve clarity and readability.***

- The description of the mediation analysis is clear. However, the interpretation of mediation as “full” versus “partial” could be reconsidered. Current methodological recommendations (e.g., Hayes, 2018) suggest avoiding this distinction, as it relies on the significance of the direct effect, which is sensitive to statistical power and not essential for establishing mediation. It may therefore be preferable to avoid this classification throughout the manuscript.
Reference:
Hayes, A. F. (2018). Introduction to mediation, moderation, and conditional process analysis: A regression-based approach (2nd ed.). Guilford Press.

***Author Response: we have revised the manuscript to remove any references to “full” or “partial” mediation. The interpretation of the mediation analysis has been updated to focus on the significance and magnitude of the indirect effect, without relying on the significance of the direct effect for classification. The corresponding sections in the Results and Discussion have been reworded accordingly.***

5) Page 10: The “Results” section could start with the paragraph on correlational analyses.
- Figure 1 appears somewhat dense; a standard correlation table (indicating positive and negative (-) correlation and significance levels with asterisks, along with a legend for p-values) may improve readability. Please also include mean and standard deviation for each variable in the table. In this case, the parts referring to the use of R could be removed.

***Author Response: Thank you for your valuable suggestion. We understand that the heatmap could be dense, so we have retained it while making improvements for better readability. To address your concerns, we have added a figure legend that includes a clear explanation of the correlations, significance levels (with asterisks for p-values). Table 2 displayed mean and SD values.***

6) Page: 12: In the “Results” section, group differences in BAS-2 are described using medians and p-values, but the corresponding test statistic is not reported. For non-parametric tests (e.g., Mann–Whitney U), reporting the test statistic alongside the p-value (and clearly referring to it in the Table 4) would improve clarity and completeness.

***Author Response: We thank the reviewer for this helpful comment. We have added a column demonstrating the test statistic in table 3***

7) Page 16: In the “Discussion” section: “These pathways may include reduced self-compassion, increased emotional avoidance, or heightened internalization of sociocultural appearance ideals.” Please add references to support these proposed alternative pathways.

***Author Response: We thank the reviewer for this suggestion. We have added supporting references to this statement.***

8) Page 17: In the “Strengths and limitations” section:
- In relation to exclusion criteria, for transparency it would be useful to clarify whether individuals with neurological disorders, psychotic conditions, or cognitive impairments were not screened for and therefore not excluded from the study.

***Author Response: We thank the reviewer for this important point. We have clarified this point.***

- Please further elaborate on the limitations associated with self-reported diagnoses.

***Author Response: We thank the reviewer for this important comment. We have clarified the implications of using self-reported psychiatric diagnoses.***

- It may also be useful to acknowledge additional unmeasured factors, such as comorbid symptoms and duration of symptoms.

***Author Response: We have acknowledged this point in the limitation section.***
