# Supplementary material for: Exploring the relationship between body appreciation and emotional regulation among Lebanese adults: The mediating role of psychological distress
Source: PLOS Glob Public Health. 2026 Jun 18;6(6):e0006725. doi: 10.1371/journal.pgph.0006725 (PMC13278471; doi:10.1371/journal.pgph.0006725)
Supplement: Attachment — Submitted filename: Reply to Reviewers.docx [file pgph.0006725.s004.docx]

**Response to Reviewers**

We would like to sincerely thank the Editor and all reviewers for their thoughtful and constructive feedback. Their comments have greatly contributed to improving the clarity, rigor, and overall quality of the manuscript. We have carefully addressed all comments and revised the manuscript accordingly. Below is a point-by-point response to each reviewer.

**Reviewer #1:** Thank you for your revisions. I have no other comments

***Author’s response:*** *We sincerely thank Reviewer #1 for taking the time to review our manuscript and positive evaluation of the revised manuscript.*

***Reviewer #2***

We would like to sincerely thank Reviewer #2 for the careful review and constructive feedback. The insightful comments have helped us further improve the manuscript.

**Comment 1:** Path b p-value inconsistency between Table 5 and Figure 2.

***Author’s Response:*** *We thank the reviewer for identifying this inconsistency. We have corrected Table 5 to ensure consistency with Figure 2, and the p-value is now reported as p < 0.001.*

**Comment 2:** Non-binary gender option should be acknowledged in the manuscript.

***Author’s Response:*** *We thank the reviewer for this important suggestion. We have now explicitly stated in the Methods section that gender was assessed as a dichotomous variable (male/female). We have also added a limitation noting that the absence of non-binary or “prefer not to say” options limits the inclusion and interpretation of gender-diverse individuals.*

**Comment 3:** Demographic skew should be included in the Abstract.

***Author’s Response:*** *We thank the reviewer for this suggestion. We have added a brief description of sample characteristics (including gender distribution, education level, and mean age) in the Methods sentence of the Abstract to improve transparency and avoid overgeneralization.*

***Comment 4:*** A small softening — e.g., "preliminary evidence that these mechanisms may also be relevant in this context, pending replication in more representative Lebanese samples" — would better align the Discussion with the limitations now acknowledged.

***Author’s Response:*** *We thank the reviewer for this valuable comment. We have revised the statement to reflect a more cautious interpretation, indicating that the findings provide preliminary evidence and should be confirmed in more representative samples.*

***Reviewer #3***

We sincerely thank Reviewer #3 for the detailed and constructive comments, which have helped improve the structure and clarity of the manuscript.

**Comment 1:** Sample section structure and content.

***Author’s Response:*** *We thank the reviewer for this helpful suggestion. We have revised the Sample section accordingly by removing the opening sentence and starting directly with study design, eligibility criteria, sample size and participant characteristics. We have also relocated descriptive sample-related information to improve clarity and distinction between sample description and study outcomes. Additionally, we corrected inconsistencies in age reporting and reduced redundancy by referring to Table 1 for demographic details.*

**Comment 2:** Results section structure and demographic redundancy.

***Author’s Response:*** *We thank the reviewer for this suggestion. We have revised the Results section to begin directly with correlational analyses, while relocating descriptive sample information to the Sample section. We have also reduced redundancy by avoiding repetition of demographic details already presented in Table 1.*

***Comment 3:*** *Statistical test labeling in Tables 2 and 3.*

***Author’s Response:*** *We thank the reviewer for this helpful suggestion. We have revised Tables 2 and 3 by replacing the generic label “test statistic” with the specific tests used (Mann–Whitney U test and Kruskal–Wallis H test), improving clarity and interpretability.*

***Comment 4:*** *Correlation heatmap presentation.*

***Response:*** *We thank the reviewer for this observation. While we agree that a traditional correlation table is more conventional, we have retained the heatmap format as it facilitates visual interpretation of relationships among variables. We appreciate the reviewer’s comment and believe the current presentation remains appropriate.*

*Top of Form*

*Bottom of Form*
