# Supplementary material for: Exploring the relationship between body appreciation and emotional regulation among Lebanese adults: The mediating role of psychological distress
Source: PLOS Glob Public Health. 2026 Jun 18;6(6):e0006725. doi: 10.1371/journal.pgph.0006725 (PMC13278471; doi:10.1371/journal.pgph.0006725)
Supplement: Peer review history

# Peer review: Decision Letter 0

**DOI:** 10.1371/journal.pgph.0006725.r001

**Authors:** Steve Zimmerman

15 Apr 2026

PGPH-D-26-00697

Exploring the Relationship between Body Appreciation and Emotional Regulation Among Lebanese Adults: The Mediating Role of Psychological Distress

PLOS Global Public Health

Dear Dr. AbouAbbas,

Thank you for submitting your manuscript to PLOS Global Public Health. After careful consideration, we feel that it has merit but does not fully meet PLOS Global Public Health’s publication criteria as it currently stands. Therefore, we invite you to submit a revised version of the manuscript that addresses the points raised during the review process.

The manuscript has been evaluated by three reviewers, and their comments are available below.

Could you please revise the manuscript to carefully address the concerns raised? (Please note that you are not required to add any requested citations).

Please submit your revised manuscript by May 30 2026 11:59PM. If you will need more time than this to complete your revisions, please reply to this message or contact the journal office at globalpubhealth@plos.org. When you're ready to submit your revision, log on to https://www.editorialmanager.com/pgph/ and select the 'Submissions Needing Revision' folder to locate your manuscript file.

Please include the following items when submitting your revised manuscript:

- A letter that responds to each point raised by the editor and reviewer(s). You should upload this letter as a separate file labeled 'Response to Reviewers'.
- A marked-up copy of your manuscript that highlights changes made to the original version. You should upload this as a separate file labeled 'Revised Manuscript with Track Changes'.
- An unmarked version of your revised paper without tracked changes. You should upload this as a separate file labeled 'Manuscript'.

Guidelines for resubmitting your figure files are available below the reviewer comments at the end of this letter.

As the corresponding author, your ORCID iD is verified in the submission system and will appear in the published article. PLOS supports the use of ORCID, and we encourage all coauthors to register for an ORCID iD and use it as well. Please encourage your coauthors to verify their ORCID iD within the submission system before final acceptance, as unverified ORCID iDs will not appear in the published article. *Only*  the individual author can complete the verification step; PLOS staff *cannot*  verify ORCID iDs on behalf of authors.

We look forward to receiving your revised manuscript.

Kind regards,

Steve Zimmerman, PhD

PLOS Staff Editor

**Journal Requirements:**

1. During your revisions, please note that a simple title correction is required: Please remove “Tittle” from the title of the manuscript that you have submitted in the online submission form. Please ensure this is updated in the manuscript file and the online submission information.

2. Please ensure that your Ethics Statement is available in its entirety at the beginning of your Methods section, under a subheading 'Ethics Statement'. It must include:

i) The name(s) of the Institutional Review Board(s) or Ethics Committee(s)

ii) The approval number(s), or a statement that approval was granted by the named board(s)

iii) (for human participants or donors) - A statement that formal consent was obtained (must state whether verbal/written) OR the reason consent was not obtained (e.g., anonymity).

vi) (for child participants) - Please update your Ethics Statement with confirmation that written informed consent was obtained from the parent/guardian of each participant under 18 years of age.

3. We note that there is identifying data in the Supporting Information file <MH Dataset.sav>. Due to the inclusion of these potentially identifying data, we have removed this file from your file inventory. Prior to sharing human research participant data, authors should consult with an ethics committee to ensure data are shared in accordance with participant consent and all applicable local laws.

Data sharing should never compromise participant privacy. It is therefore not appropriate to publicly share personally identifiable data on human research participants. The following are examples of data that should not be shared:

-Name, initials, physical address

-Ages more specific than whole numbers

-Internet protocol (IP) address

-Specific dates (birth dates, death dates, examination dates, etc.)

-Contact information such as phone number or email address

-Location data

-ID numbers that seem specific (long numbers, include initials, titled “Hospital ID”) rather than random (small numbers in numerical order)

Data that are not directly identifying may also be inappropriate to share, as in combination they can become identifying. For example, data collected from a small group of participants, vulnerable populations, or private groups should not be shared if they involve indirect identifiers (such as sex, ethnicity, location, etc.) that may risk the identification of study participants.

Additional guidance on preparing raw data for publication can be found in our Data Policy (https://journals.plos.org/plosone/s/data-availability#loc-human-research-participant-data-and-other-sensitive-data) and in the following article: http://www.bmj.com/content/340/bmj.c181.long.

Please remove or anonymize all personal information (<specific identifying information in file to be removed>), ensure that the data shared are in accordance with participant consent, and re-upload a fully anonymized data set. Please note that spreadsheet columns with personal information must be removed and not hidden as all hidden columns will appear in the published file.

4. We have noticed that you have uploaded Supporting Information files, but you have not included a list of legends. Please add a full list of legends for your Supporting Information files after the references list.

If the reviewer comments include a recommendation to cite specific previously published works, please review and evaluate these publications to determine whether they are relevant and should be cited. There is no requirement to cite these works unless the editor has indicated otherwise.

**Additional Editor Comments (if provided):**

[Note: HTML markup is below. Please do not edit.]

Reviewers' comments:

Reviewer's Responses to Questions

**Comments to the Author**

1. Does this manuscript meet PLOS Global Public Health’s publication criteria? Is the manuscript technically sound, and do the data support the conclusions? The manuscript must describe methodologically and ethically rigorous research with conclusions that are appropriately drawn based on the data presented.

Reviewer #1: Yes

Reviewer #2: Yes

Reviewer #3: Yes

**********

2. Has the statistical analysis been performed appropriately and rigorously?

Reviewer #1: Yes

Reviewer #2: Yes

Reviewer #3: Yes

**********

3. Have the authors made all data underlying the findings in their manuscript fully available (please refer to the Data Availability Statement at the start of the manuscript PDF file)?

The PLOS Data policy requires authors to make all data underlying the findings described in their manuscript fully available without restriction, with rare exception. The data should be provided as part of the manuscript or its supporting information, or deposited to a public repository. For example, in addition to summary statistics, the data points behind means, medians and variance measures should be available. If there are restrictions on publicly sharing data—e.g. participant privacy or use of data from a third party—those must be specified.

Reviewer #1: No

Reviewer #2: Yes

Reviewer #3: Yes

**********

4. Is the manuscript presented in an intelligible fashion and written in standard English?

PLOS Global Public Health does not copyedit accepted manuscripts, so the language in submitted articles must be clear, correct, and unambiguous. Any typographical or grammatical errors should be corrected at revision, so please note any specific errors here.

Reviewer #1: Yes

Reviewer #2: Yes

Reviewer #3: Yes

**********

5. Review Comments to the Author

Please use the space provided to explain your answers to the questions above. You may also include additional comments for the author, including concerns about dual publication, research ethics, or publication ethics. (Please upload your review as an attachment if it exceeds 20,000 characters)

**Reviewer #1** : Dear authors,

thank you for your contribution. Here are my suggestions

- Introduction: please, foster your background with further details regarding psychological constructs (see: Ongaro et al., 2021) and related interventions. For example, what about how to address these points? Please, see: Sebri et al., 2020; 2023. Second, please, structure your hypothesis and goals better

- Methods: the recruitment process should be better explained

- Discussion: I would suggest proposing new approaches to this topic. As known, new technologies are currently an innovative, effective approach that could be applied, with benefits and limits. Please, discuss this point, considering your research study and findings (see: Triberti et al., 2021).

References

- Triberti, S., Petrella, F., Gorini, A., Pappalardo, O., Sebri, V., Savioni, L., ... & Pravettoni, G. (2021). Augmenting surgery: medical students’ assessment and ergonomics of 3d holograms vs. CT scans for pre-operative planning. EAI Endorsed Transactions on Pervasive Health and Technology, 7(25).

- Ongaro, G., Cincidda, C., Sebri, V., Savioni, L., Triberti, S., Ferrucci, R., ... & Pravettoni, G. (2021). A 6-month follow-up study on worry and its impact on well-being during the first wave of COVID-19 pandemic in an Italian sample. Frontiers in Psychology, 12, 703214.

- Sebri, V., Savioni, L., Triberti, S., Durosini, I., Mazzocco, K., & Pravettoni, G. (2020). Do you transfer your skills? From sports to health management in cancer patients. Frontiers in Psychology, 11, 546.

- Sebri, V., Durosini, I., Strika, M., Pizzoli, S. F. M., Mazzocco, K., & Pravettoni, G. (2023). Virtual reality for the promotion of interoception awareness and body image in breast cancer survivors: a study protocol. Frontiers in Psychology, 14, 1165905.

**Reviewer #2:** The manuscript addresses a timely and culturally relevant question: whether psychological distress mediates the association between emotion regulation difficulties and body appreciation in Lebanese adults. The topic is well-positioned within the growing literature on positive body image and contributes to the understudied non-Western context. The study design is appropriate, the sample size exceeds the a priori power calculation by a wide margin (N = 682 vs. 103 required), and the statistical approach (PROCESS Model 4 with bootstrapping) is sound. The validated instruments (BAS-2, DERS-16, BDS-10) are culturally appropriate, all having Arabic-language validation studies conducted in Lebanese samples. The writing is generally clear, and the structure follows standard IMRAD format.

However, several methodological and presentational issues must be addressed before the manuscript can be recommended for publication. These range from a substantive citation error and inconsistencies in reported statistics to gaps in the methodological description and underexplored aspects of the discussion. None of these issues are fatal, but they require careful attention.

Misattribution of the BAS-2 source citation

At line 167, the manuscript describes the BAS-2 and cites reference [20] (Gratz & Roemer, 2004) as the source. Reference [20] is the original development paper for the DERS, not the BAS-2. The correct citation for the BAS-2 is reference [7] (Tylka & Wood-Barcalow, 2015). This error also appears at line 173, where [20] is correctly cited for the DERS. The dual citation of the same reference for two different instruments is confusing and must be corrected: line 167 should cite [7], not [20].

Discrepancy in Table 4 (Mental Health Disorder medians)

Table 4 reports that participants with “Any Mental Health Disorder = No” have a median BAS-2 of 37 (IQR 15), while those with “Yes” have a median of 40 (IQR 11). This is counterintuitive — it suggests that individuals reporting a mental health disorder have higher body appreciation than those without one. However, the text at lines 277–278 states the opposite: “Participants reporting any mental health disorder had significantly lower body appreciation compared to those without a disorder (Median = 40 vs. 37; p-value = 0.001).” One of these is incorrect. Given that the text assigns Median = 40 to those without a disorder and 37 to those with one, the table rows for “No” and “Yes” appear to be inverted. The authors must verify the raw data and correct either the table or the text.

Convenience sampling and generalizability

The sample is 73.9% female, 87.5% university-educated, and predominantly young (mean age 27.5). The convenience sampling strategy via social media introduces considerable selection bias. While the authors acknowledge this in the Limitations section, the Discussion interprets results as if they generalize to “Lebanese adults” broadly. The cultural generalizations about Lebanon (lines 387–393) should be tempered, and the title itself (“Among Lebanese Adults”) could include a qualifier (e.g., “predominantly young and educated”). The Discussion should explicitly state that the demographic skew limits inferences about the broader Lebanese population.

Covariates inconsistency between regression and mediation

The multiple regression model (Table 5) includes six predictors: DERS-16, BDS-10, age, gender, BMI, and self-reported mental health disorder. However, the mediation analysis (lines 215–217) states that covariates included “age, gender, BMI, presence of mental health disorder, marital status, education, occupation, income, and governorate.” This means the mediation model controlled for five additional covariates (marital status, education, occupation, income, governorate) that were absent from the regression model. The authors should explain why the two models used different covariate sets, or ideally harmonize them. If the covariates were intentionally different, justification is needed.

Missing non-binary or non-response gender option

Gender is coded dichotomously (male/female). The manuscript does not mention whether a non-binary or “prefer not to say” option was offered. Given that body image research is increasingly sensitive to gender diversity, the authors should clarify the gender categories available in the survey and acknowledge this as a limitation if no non-binary option was offered.

Typo issues

“Tittle” appears at line 1 of the manuscript body. Should be “Title.”

Sentence fragment at lines 115–118: “The present study aims to address these gaps by examining the mediating role of psychological distress in the relationship between emotion regulation and body appreciation has not been examined in Lebanese or broader Arab populations.” This sentence conflates two clauses and is grammatically broken. Suggested revision: “The present study aims to address these gaps by examining the mediating role of psychological distress in the relationship between emotion regulation and body appreciation — a mechanism that has not been examined in Lebanese or broader Arab populations.”

Lowercase “higher” at line 108: “makes them more self-critical[20]. higher distress can undermine…” — “Higher” should be capitalized as it begins a new sentence.

Missing space before citation at lines 86–87: “respecting the body”[7] should be “body” [7].

§§§

Correlation report inconsistency for BDS-10 and Age: lines 267–268 report the age–BDS-10 correlation as r = −0.11, p < 0.05, but Figure 1 (heatmap) shows this value as −0.01. The manuscript text and the figure are inconsistent. The authors must verify which value is correct.

BMI correlation with DERS-16: Figure 1 shows BMI–DERS-16 correlation as −0.05, but this is not reported in the text. For completeness, it should be noted. Also, the text (lines 267–269) reports BMI correlated with BAS-2 as r = −0.219, while Figure 1 shows −0.22 — this is consistent but the text should round to two decimal places uniformly throughout.

Monthly income data: Table 1 reports income data for n = 524, meaning 158 participants (23.2%) did not report income. This missingness is not discussed. The authors should mention this and clarify whether these participants were excluded from income-related analyses.

Ratio of mediation: The manuscript reports partial mediation but does not report the proportion of the total effect mediated (indirect/total = 0.095/0.168 = 56.5%). Including this ratio would strengthen the interpretation.

Effect size interpretation: R-squared = 0.211 means the model explains about 21% of BAS-2 variance. While the authors report this, they do not discuss whether this is a meaningful or modest amount for a model with six predictors. Contextualising this against comparable studies would be helpful.

Figure 2 inconsistency: The mediation path diagram shows Path b as p = 0.001, but Table 6 reports p < 0.001. These should be harmonised.

Bibliographic Issues

Reference [20] misattribution: Gratz & Roemer (2004) is cited for BAS-2 at line 167, but describes the DERS. Must cite [7] instead.

Reference [4]: Author surname is misspelled as “TyLka” — should be “Tylka.” Page range is listed as pp. 56–58 but likely spans pp. 56–64 (confirm against the physical volume). Chapter title is absent.

Reference [5] (DSM-5): Citation is incomplete — missing full title (Diagnostic and statistical manual of mental disorders, 5th ed.), publisher (American Psychiatric Publishing), and DOI (10.1176/appi.books.9780890425596).

Reference [29] (Hayes, PROCESS macro): The citation is incomplete. Hayes (2022) refers to Introduction to Mediation, Moderation, and Conditional Process Analysis (3rd ed., Guilford Press). The URL alone is insufficient; full book citation should be provided.

Reference [21] (Levy et al.): Year cited as 2025, but CrossRef records online publication in 2024. Verify print issue date.

References [14], [17]: Multiple co-authors omitted (Nolen-Hoeksema & Schweizer for [14]; five co-authors for [17]). While Vancouver-style numeric citations can abbreviate, PLOS journals use a full author list. Verify compliance with journal citation guidelines.

Recommendation: Minor Revision.

The manuscript makes a relevant contribution to the body image literature in a non-Western context. The study is methodologically sound in its core design, and the mediation analysis is well-executed. However, the citation error (Major Issue 2.1), the likely table inversion (Major Issue 2.2), the covariate inconsistency between models (Major Issue 2.4), and the correlation discrepancy between text and Figure 1 (Minor Issue 3.5) must be resolved before publication. These appear to be correctable errors rather than fundamental design flaws. Once addressed, the paper will be suitable for publication in PLOS Global Public Health.

**Reviewer #3:**  The paper investigates the association between emotion regulation difficulties and body appreciation among Lebanese adults, and examines the mediating role of psychological distress. The cultural context is valuable, and the manuscript is generally clear. To further improve readability, some reorganization and clarifications are suggested.

Below are my comments:

1) Page 4: In the “Introduction” section:

- After introducing emotion regulation for the first time, please indicate the abbreviation (ER) to create a clear reference for its later use in the text.

- “Despite growing evidence linking emotion regulation to negative body image outcomes, little is known about its relationship with positive body image.”

A meta-analysis reported positive associations between body appreciation and adaptive emotion regulation (see Linardon et al., 2022); it may be useful to include this data and reference.

Reference:

Linardon, J., McClure, Z., Tylka, T. L., & Fuller-Tyszkiewicz, M. (2022). Body appreciation and its psychological correlates: A systematic review and meta-analysis. Body Image, 42, 287–296.

- The statement that “most existing research on body appreciation and emotion regulation has been conducted in Western contexts” is relevant. In this regard, it may be useful to further elaborate, already in the Introduction, on how body image and body appreciation are conceptualized within the Lebanese or broader Arabic context, in order to better support the rationale for the study. This would also facilitate a clearer link with the discussion of cultural aspects later in the manuscript.

2) Page 6: The “Methods” section appears somewhat fragmented. It may be useful to consolidate and streamline sample-related information into a single paragraph, “Sample”, including the sample size (with the sample size calculation reported in a footnote), online data collection procedures, recruitment (please provide examples of social media platforms and community outreach), inclusion and exclusion criteria, and self-reported mental health disorders. A brief description of how participants received study information, provided informed consent, and that participation was voluntary would also be helpful. Referring to Table 1 for detailed socio-demographic information could avoid listing all variables in the text.

3) Page 7: The “Data collection” section could be renamed “Instruments” and structured to list the selected measures. For each instrument, please report the original validation reference followed by the Arabic validation reference, and include the internal consistency in the current sample. For the “clinical and diagnosed mental health variables,” please specify the exact questions used. As Table 2 appears to report similar information, this may be redundant, and its removal could be considered.

4) Page 8: “Statistical Analysis” section: before describing how the analyses were performed, please indicate whether all measures were completed and whether any validity checks (e.g., response time, attention filters) were used to identify careless responders or bots.

- Please also indicate how potential outliers were examined and handled.

- The statement “for continuous variables that were approximately normally distributed” is somewhat vague; please clarify how normality was assessed (e.g., Shapiro–Wilk test).

- Please report Tolerance and VIF values to address potential multicollinearity.

- Before describing the mediation analysis, please specify the types of correlations computed and the statistical tests used to compare participants with and without mental health disorders.

- Throughout the manuscript, referring to constructs rather than instrument names may improve readability, both in the text and in the tables.

- The description of the mediation analysis is clear. However, the interpretation of mediation as “full” versus “partial” could be reconsidered. Current methodological recommendations (e.g., Hayes, 2018) suggest avoiding this distinction, as it relies on the significance of the direct effect, which is sensitive to statistical power and not essential for establishing mediation. It may therefore be preferable to avoid this classification throughout the manuscript.

Reference:

Hayes, A. F. (2018). Introduction to mediation, moderation, and conditional process analysis: A regression-based approach (2nd ed.). Guilford Press.

5) Pag 10: The “Results” section could start with the paragraph on correlational analyses.

- Figure 1 appears somewhat dense; a standard correlation table (indicating positive and negative (-) correlation and significance levels with asterisks, along with a legend for p-values) may improve readability. Please also include mean and standard deviation for each variable in the table. In this case, the parts referring to the use of R could be removed.

6) Page: 12: In the “Results” section, group differences in BAS-2 are described using medians and p-values, but the corresponding test statistic is not reported. For non-parametric tests (e.g., Mann–Whitney U), reporting the test statistic alongside the p-value (and clearly referring to it in the Table 4) would improve clarity and completeness.

7) Page 16: In the “Discussion” section:

“These pathways may include reduced self-compassion, increased emotional avoidance, or heightened internalization of sociocultural appearance ideals.”

Please add references to support these proposed alternative pathways.

8) Page 17: In the “Strengths and limitations” section:

- In relation to exclusion criteria, for transparency it would be useful to clarify whether individuals with neurological disorders, psychotic conditions, or cognitive impairments were not screened for and therefore not excluded from the study.

- Please further elaborate on the limitations associated with self-reported diagnoses.

- It may also be useful to acknowledge additional unmeasured factors, such as comorbid symptoms and duration of symptoms.

**********

6. PLOS authors have the option to publish the peer review history of their article (what does this mean?). If published, this will include your full peer review and any attached files.

**Do you want your identity to be public for this peer review?**  If you choose “no”, your identity will remain anonymous but your review may still be made public.

For information about this choice, including consent withdrawal, please see our Privacy Policy.

Reviewer #1: No

Reviewer #2: **Yes:** Francesco Ernesto Alessi Longa

Reviewer #3: No

**********

[NOTE: If reviewer comments were submitted as an attachment file, they will be attached to this email and accessible via the submission site. Please log into your account, locate the manuscript record, and check for the action link "View Attachments". If this link does not appear, there are no attachment files.]

**Figure Resubmissions:**

While revising your submission, we strongly recommend that you use PLOS’s NAAS tool (https://ngplosjournals.pagemajik.ai/artanalysis) to test your figure files. NAAS can convert your figure files to the TIFF file type and meet basic requirements (such as print size, resolution), or provide you with a report on issues that do not meet our requirements and that NAAS cannot fix.

After uploading your figures to PLOS’s NAAS tool - https://ngplosjournals.pagemajik.ai/artanalysis, NAAS will process the files provided and display the results in the "Uploaded Files" section of the page as the processing is complete. If the uploaded figures meet our requirements (or NAAS is able to fix the files to meet our requirements), the figure will be marked as "fixed" above. If NAAS is unable to fix the files, a red "failed" label will appear above. When NAAS has confirmed that the figure files meet our requirements, please download the file via the download option, and include these NAAS processed figure files when submitting your revised manuscript.

# Author comment: Author response to Decision Letter 1

**DOI:** 10.1371/journal.pgph.0006725.r002

28 Apr 2026

Attachment Submitted filename: Response to Reviewers.docx

# Peer review: Decision Letter 1

**DOI:** 10.1371/journal.pgph.0006725.r003

**Authors:** Steve Zimmerman, Katherine Kokkinias

14 May 2026

PGPH-D-26-00697R1

Exploring the Relationship between Body Appreciation and Emotional Regulation Among Lebanese Adults: The Mediating Role of Psychological Distress

PLOS Global Public Health

Dear Dr. AbouAbbas,

Thank you for submitting your manuscript to PLOS Global Public Health. After careful consideration, we feel that it has merit but does not fully meet PLOS Global Public Health’s publication criteria as it currently stands. Therefore, we invite you to submit a revised version of the manuscript that addresses the points raised during the review process.

==============================

The manuscript has been evaluated by three reviewers, and their comments are available below.

The reviewers have raised a number of concerns that need attention. They provide suggestions to improve the clarity of the manuscript. They also highlight discrepancies that should be addressed in the results and highlight limitations that should be described explicitly.

Could you please revise the manuscript to carefully address the concerns raised?

==============================

Please submit your revised manuscript by Jun 13 2026 11:59PM. If you will need more time than this to complete your revisions, please reply to this message or contact the journal office at globalpubhealth@plos.org. When you're ready to submit your revision, log on to https://www.editorialmanager.com/pgph/ and select the 'Submissions Needing Revision' folder to locate your manuscript file.

Please include the following items when submitting your revised manuscript:

- A letter that responds to each point raised by the editor and reviewer(s). You should upload this letter as a separate file labeled 'Response to Reviewers'.
- A marked-up copy of your manuscript that highlights changes made to the original version. You should upload this as a separate file labeled 'Revised Manuscript with Track Changes'.
- An unmarked version of your revised paper without tracked changes. You should upload this as a separate file labeled 'Manuscript'.

Guidelines for resubmitting your figure files are available below the reviewer comments at the end of this letter.

As the corresponding author, your ORCID iD is verified in the submission system and will appear in the published article. PLOS supports the use of ORCID, and we encourage all coauthors to register for an ORCID iD and use it as well. Please encourage your coauthors to verify their ORCID iD within the submission system before final acceptance, as unverified ORCID iDs will not appear in the published article. *Only*  the individual author can complete the verification step; PLOS staff *cannot*  verify ORCID iDs on behalf of authors.

We look forward to receiving your revised manuscript.

Kind regards,

Katherine Demi Kokkinias, Ph.D.

Staff Editor

PLOS Global Public Health

**Journal Requirements:**

If the reviewer comments include a recommendation to cite specific previously published works, please review and evaluate these publications to determine whether they are relevant and should be cited. There is no requirement to cite these works unless the editor has indicated otherwise.

**Additional Editor Comments (if provided):**

[Note: HTML markup is below. Please do not edit.]

Reviewers' comments:

Reviewer's Responses to Questions

**Comments to the Author**

1. If the authors have adequately addressed your comments raised in a previous round of review and you feel that this manuscript is now acceptable for publication, you may indicate that here to bypass the “Comments to the Author” section, enter your conflict of interest statement in the “Confidential to Editor” section, and submit your "Accept" recommendation.

Reviewer #1: (No Response)

Reviewer #2: All comments have been addressed

Reviewer #3: (No Response)

**********

2. Does this manuscript meet PLOS Global Public Health’s publication criteria? Is the manuscript technically sound, and do the data support the conclusions? The manuscript must describe methodologically and ethically rigorous research with conclusions that are appropriately drawn based on the data presented.

Reviewer #1: (No Response)

Reviewer #2: Yes

Reviewer #3: Yes

**********

3. Has the statistical analysis been performed appropriately and rigorously?

Reviewer #1: (No Response)

Reviewer #2: Yes

Reviewer #3: Yes

**********

4. Have the authors made all data underlying the findings in their manuscript fully available (please refer to the Data Availability Statement at the start of the manuscript PDF file)?

The PLOS Data policy requires authors to make all data underlying the findings described in their manuscript fully available without restriction, with rare exception. The data should be provided as part of the manuscript or its supporting information, or deposited to a public repository. For example, in addition to summary statistics, the data points behind means, medians and variance measures should be available. If there are restrictions on publicly sharing data—e.g. participant privacy or use of data from a third party—those must be specified.

Reviewer #1: (No Response)

Reviewer #2: Yes

Reviewer #3: Yes

**********

5. Is the manuscript presented in an intelligible fashion and written in standard English?

PLOS Global Public Health does not copyedit accepted manuscripts, so the language in submitted articles must be clear, correct, and unambiguous. Any typographical or grammatical errors should be corrected at revision, so please note any specific errors here.

Reviewer #1: (No Response)

Reviewer #2: Yes

Reviewer #3: Yes

**********

6. Review Comments to the Author

Please use the space provided to explain your answers to the questions above. You may also include additional comments for the author, including concerns about dual publication, research ethics, or publication ethics. (Please upload your review as an attachment if it exceeds 20,000 characters)

**Reviewer #1:**  Thank you for your revisions. I have no other comments

**Reviewer #2:**  I thank the authors for their careful and thorough response to my previous comments. The four major issues I had flagged in the first round have all been satisfactorily resolved.

A small number of residual issues remain, none of which are substantive but which should be addressed before acceptance.

1. Path b p-value inconsistency between Table 5 and Figure 2. Figure 2 has been correctly updated to "−0.311, p < 0.001", but Table 5 still reports "0.001" for the same path. Since this is the same coefficient as the BDS-10 → BAS-2 effect in Table 4 (where it appears as p < 0.001), Table 5 should be harmonised to "< 0.001".

2. Non-binary gender option — limitation should appear in the manuscript itself. The authors' explanation in the rebuttal letter (dichotomous coding reflecting the Lebanese cultural context) is reasonable, but it currently lives only in the response. To ensure transparency, the Methods should state explicitly that gender was assessed as a dichotomous variable, and the Strengths and Limitations section should acknowledge that the absence of a non-binary or "prefer not to say" option limits inferences about gender-diverse individuals.

3. Demographic skew should also be flagged in the Abstract. Since the authors have decided to retain the original title (which is reasonable), I recommend adding a brief descriptor of sample composition to the Methods sentence of the Abstract (e.g., "…(N = 682; 73.9% female, 87.5% with university-level education, mean age 27.5 years)…") to prevent over-generalisation by readers who only consult the abstract.

4. Minor attenuation in the Discussion. The statement at lines 392–394 that "the mechanisms linking emotion regulation, distress, and body appreciation observed in Western samples are also relevant in this context" is still phrased as a relatively confident inference. A small softening — e.g., "preliminary evidence that these mechanisms may also be relevant in this context, pending replication in more representative Lebanese samples" — would better align the Discussion with the limitations now acknowledged.

Recommendation: Minor revision. All substantive concerns from my previous review have been addressed. The residual issues are surface-level corrections that should not require any new analyses. Once resolved, the manuscript will, in my view, be suitable for publication in PLOS Global Public Health

Reviewer #3: In general, the authors have appropriately addressed the requested revisions. A few remaining minor points are listed below:

1) The opening sentence of the “Sample” section:

“A cross-sectional design was employed to examine the relationship between body appreciation and emotional regulation, with psychological distress as a potential mediator among adults”

does not belong in this section, as it refers to the study design and analytical approach. Moreover, this information is already appropriately reported in the Statistical Analysis section. It would therefore be preferable to remove it from the “Sample” section.

I would suggest that the “Sample” section be revised to start directly with sample size and characteristics. A possible structure would be:

"A total of 692 responses were collected, of which 10 participants were excluded for being younger than 18 years, resulting in a final sample of 682 eligible participants (see Table 1 for sample characteristics). Among the participants, 124 (18.2%) reported having a mental health disorder. The most commonly reported disorders were anxiety (75; 11.0%) and depression (62; 9.1%), followed by eating disorders (6; 0.9%), bipolar disorder (5; 0.7%), and obsessive-compulsive disorder (OCD; 1; 0.1%)"

The section could then continue as revised from “Data were collected over a period of 4 months…” to the end. This would improve clarity by presenting sample size and composition at the beginning of the section and enhancing the overall structure of the section.

2) I would suggest that the “Results” section be revised to start directly with the paragraph on correlational analyses, as previously suggested. While the main content of “Baseline characteristics of the study participants” and “Self-reported mental health disorders” would be more appropriately reported in the “Sample” section (see point 1), as they describe the sample rather than study outcomes.

In addition, there appears to be a discrepancy in the reported age statistics: the text reports a mean age of 27.5 (SD = 9.1), whereas the corresponding values in Table 1 differ and require correction.

Finally, the detailed description of gender and other demographic variables in the text is somewhat redundant, as these are already clearly presented in Table 1; referring to the table would improve conciseness and readability.

3) The statistical analysis section is now clear. However, for clarity, it would be preferable to specify the exact statistic used in the tables 2 and 3 rather than using the generic label “test statistic”, as this would allow readers to clearly interpret the reported values.

4) The revisions improve the readability of the heatmap of correlations. In my opinion, a traditional correlation table would generally be more standard and less dense; however, the current presentation is acceptable.

**********

7. PLOS authors have the option to publish the peer review history of their article (what does this mean?). If published, this will include your full peer review and any attached files.

**Do you want your identity to be public for this peer review?**  If you choose “no”, your identity will remain anonymous but your review may still be made public.

For information about this choice, including consent withdrawal, please see our Privacy Policy.

Reviewer #1: No

Reviewer #2: **Yes:** Francesco Ernesto Alessi Longa

Reviewer #3: No

**********

[NOTE: If reviewer comments were submitted as an attachment file, they will be attached to this email and accessible via the submission site. Please log into your account, locate the manuscript record, and check for the action link "View Attachments". If this link does not appear, there are no attachment files.]

**Figure Resubmissions:**

While revising your submission, we strongly recommend that you use PLOS’s NAAS tool (https://ngplosjournals.pagemajik.ai/artanalysis) to test your figure files. NAAS can convert your figure files to the TIFF file type and meet basic requirements (such as print size, resolution), or provide you with a report on issues that do not meet our requirements and that NAAS cannot fix.

After uploading your figures to PLOS’s NAAS tool - https://ngplosjournals.pagemajik.ai/artanalysis, NAAS will process the files provided and display the results in the "Uploaded Files" section of the page as the processing is complete. If the uploaded figures meet our requirements (or NAAS is able to fix the files to meet our requirements), the figure will be marked as "fixed" above. If NAAS is unable to fix the files, a red "failed" label will appear above. When NAAS has confirmed that the figure files meet our requirements, please download the file via the download option, and include these NAAS processed figure files when submitting your revised manuscript.

# Author comment: Author response to Decision Letter 2

**DOI:** 10.1371/journal.pgph.0006725.r004

15 May 2026

Attachment Submitted filename: Reply to Reviewers.docx

# Peer review: Decision Letter 2

**DOI:** 10.1371/journal.pgph.0006725.r005

**Authors:** Steve Zimmerman, Katherine Kokkinias, Julia Robinson

7 Jun 2026

Exploring the Relationship between Body Appreciation and Emotional Regulation Among Lebanese Adults: The Mediating Role of Psychological Distress

PGPH-D-26-00697R2

Dear Dr AbouAbbas,

We are pleased to inform you that your manuscript 'Exploring the Relationship between Body Appreciation and Emotional Regulation Among Lebanese Adults: The Mediating Role of Psychological Distress' has been provisionally accepted for publication in PLOS Global Public Health.

Please note it appears that Reviewer 1 re-submitted previous comments in error. You can feel free to disregard these.

Before your manuscript can be formally accepted you will need to complete some formatting changes, which you will receive in a follow up email. A member of our team will be in touch with a set of requests.

Please note that your manuscript will not be scheduled for publication until you have made the required changes, so a swift response is appreciated.

IMPORTANT: The editorial review process is now complete. PLOS will only permit corrections to spelling, formatting or significant scientific errors from this point onwards. Requests for major changes, or any which affect the scientific understanding of your work, will cause delays to the publication date of your manuscript.

If your institution or institutions have a press office, please notify them about your upcoming paper to help maximize its impact. If they'll be preparing press materials, please inform our press team as soon as possible -- no later than 48 hours after receiving the formal acceptance. Your manuscript will remain under strict press embargo until 2 pm Eastern Time on the date of publication. For more information, please contact globalpubhealth@plos.org.

Thank you again for supporting Open Access publishing; we are looking forward to publishing your work in PLOS Global Public Health.

Best regards,

Julia Robinson

Staff Editor

PLOS Global Public Health

***********************************************************

Reviewer Comments (if any, and for reference):

Reviewer's Responses to Questions

**Comments to the Author**

1. If the authors have adequately addressed your comments raised in a previous round of review and you feel that this manuscript is now acceptable for publication, you may indicate that here to bypass the “Comments to the Author” section, enter your conflict of interest statement in the “Confidential to Editor” section, and submit your "Accept" recommendation.

Reviewer #1: All comments have been addressed

Reviewer #2: All comments have been addressed

Reviewer #3: All comments have been addressed

**********

2. Does this manuscript meet PLOS Global Public Health’s publication criteria? Is the manuscript technically sound, and do the data support the conclusions? The manuscript must describe methodologically and ethically rigorous research with conclusions that are appropriately drawn based on the data presented.

Reviewer #1: Yes

Reviewer #2: Yes

Reviewer #3: Yes

**********

3. Has the statistical analysis been performed appropriately and rigorously?

Reviewer #1: Yes

Reviewer #2: Yes

Reviewer #3: Yes

**********

4. Have the authors made all data underlying the findings in their manuscript fully available (please refer to the Data Availability Statement at the start of the manuscript PDF file)?

The PLOS Data policy requires authors to make all data underlying the findings described in their manuscript fully available without restriction, with rare exception. The data should be provided as part of the manuscript or its supporting information, or deposited to a public repository. For example, in addition to summary statistics, the data points behind means, medians and variance measures should be available. If there are restrictions on publicly sharing data—e.g. participant privacy or use of data from a third party—those must be specified.

Reviewer #1: Yes

Reviewer #2: Yes

Reviewer #3: Yes

**********

5. Is the manuscript presented in an intelligible fashion and written in standard English?

PLOS Global Public Health does not copyedit accepted manuscripts, so the language in submitted articles must be clear, correct, and unambiguous. Any typographical or grammatical errors should be corrected at revision, so please note any specific errors here.

Reviewer #1: (No Response)

Reviewer #2: Yes

Reviewer #3: Yes

**********

6. Review Comments to the Author

Please use the space provided to explain your answers to the questions above. You may also include additional comments for the author, including concerns about dual publication, research ethics, or publication ethics. (Please upload your review as an attachment if it exceeds 20,000 characters)

**Reviewer #1:**  Dear authors,

thank you for your contribution. Here are my suggestions

- Introduction: please, foster your background with further details regarding psychological constructs and related interventions. For example, what about how to address these points? Please, see: Sebri et al., 2024a,b,c. Second, please, structure your hypothesis and goals better

- Methods: the recruitment process should be better explained

- Discussion: I would suggest proposing new approaches to this topic. As known, new technologies are currently an innovative, effective approach that could be applied, with benefits and limits. Please, discuss this point, considering your research study and findings (see: Triberti et al., 2021).

References

- Triberti, S., Petrella, F., Gorini, A., Pappalardo, O., Sebri, V., Savioni, L., ... & Pravettoni, G. (2021). Augmenting surgery: medical students’ assessment and ergonomics of 3d holograms vs. CT scans for pre-operative planning. EAI Endorsed Transactions on Pervasive Health and Technology, 7(25).

- Sebri, V., Marzorati, C., Dorangricchia, P., Monzani, D., Grasso, R., Prelaj, A., ... & Pravettoni, G. (2024a). The impact of decision tools during oncological consultation with lung cancer patients: A systematic review within the I3LUNG project. Cancer Medicine, 13(9), e7159.

- Sebri, V., Policardo, G. R., & Pravettoni, G. (2024b). How to improve emotional regulation in breast cancer survivors? A psychological intervention. Frontiers in Psychology, 15, 1443635.

- Sebri, V., Pizzoli, S. F. M., Marzorati, C., Mazzocco, K., & Pravettoni, G. (2024c). Interoception and body image in breast cancer patients: a mindfulness-based stress reduction protocol. Frontiers in Psychology, 15, 1394355.

**Reviewer #2:**  The rebuttal letter states that the Methods section has been updated to explicitly note that gender was assessed as a dichotomous variable (male/female). The corresponding limitation has indeed been added to the Strengths and Limitations section (lines 415–417), and I thank the authors for this.

However, in the Methods, the Socio-demographic information item (line 191) still lists the variables as: “age, gender, marital status, educational level, occupation, and income”, without specifying the dichotomous coding of gender. To match what is reported in the rebuttal and to ensure full methodological transparency at the point where the variable is introduced, I would suggest a brief addition such as:

“…age, gender (assessed dichotomously as male/female), marital status, educational level, occupation, and income.”

This is a one-line edit and does not require any new analysis.

**Reviewer #3:**  (No Response)

**********

7. PLOS authors have the option to publish the peer review history of their article (what does this mean?). If published, this will include your full peer review and any attached files.

**Do you want your identity to be public for this peer review?**  If you choose “no”, your identity will remain anonymous but your review may still be made public.

For information about this choice, including consent withdrawal, please see our Privacy Policy.

Reviewer #1: No

Reviewer #2: **Yes:** Francesco Ernesto Alessi Longa

Reviewer #3: No

**********
